# SGIP1α functions as a selective endocytic adaptor for the internalization of synaptotagmin 1 at synapses

**DOI:** 10.1186/s13041-019-0464-1

**Published:** 2019-05-03

**Authors:** Sang-Eun Lee, Soomin Jeong, Unghwi Lee, Sunghoe Chang

**Affiliations:** Department of Physiology and Biomedical Sciences, Neuroscience Research Institute, Seoul National University College of Medicine, Seoul, 03080 South Korea

**Keywords:** SGIP1α, synaptotagmin 1, Synaptic vesicle, Clathrin-mediated endocytosis

## Abstract

Proper sorting of exocytosed synaptic vesicle (SV) proteins into individual SVs during endocytosis is of the utmost importance for the fidelity of subsequent neurotransmission. Recent studies suggest that each SV protein is sorted into individual SVs by its own dedicated adaptors as well as by association between SV proteins. The SH3-containing GRB2-like protein 3-interacting protein 1 (SGIP1), an ortholog of Fer/Cip4 homology domain-only (FCHo) proteins, contains a μ-homology domain (μHD) and binds AP-2 and Eps15, thus functioning as an endocytic regulator of clathrin-mediated endocytosis (CME). Its longest isoform SGIP1α is predominantly expressed in the brain but the functional significance of SGIP1 in SV recycling remains unknown. Here, we found that SGIP1α, a brain-specific long isoform of SGIP1 binds synaptotagmin1 (Syt1) via its μHD and promotes the internalization of Syt1 on the neuronal surface. The small hairpin RNA (shRNA)-mediated knockdown (KD) of SGIP1α caused selective impairment of Syt1 internalization at hippocampal synapses and it was fully rescued by coexpression of the shRNA-resistant form of SGIP1α in KD neurons. We further found that the μHD of SGIP1α is structurally similar to those of AP-2 and stonin2, and mutations at Trp771 and Lys781, which correspond to Syt1-recognition motifs of AP-2 and stonin2, to Ala bound less efficiently to Syt1 and failed to rescue the endocytic defect of Syt1 caused by KD. Our results indicate that SGIP1α is an endocytic adaptor dedicated to the retrieval of surface-stranded Syt1. Since endocytic sorting of Syt1 is also mediated by the overlapping activities of synaptic vesicle glycoprotein 2A/B (SV2A/B) and stonin2, our results suggest that complementary fail-safe mechanism by these proteins ensures high fidelity of Syt1 retrieval.

## Introduction

Neurotransmission involves the calcium (Ca^2+^)-regulated fusion of SVs, a process that requires the Ca^2+^ sensor Syt1 at the active zone, which defines sites of neurotransmitter release [1]. Post-exocytic SV proteins are retrieved by endocytosis from the plasma membrane to regenerate new SVs [2]. More than forty different integral membrane proteins that are essential for SV trafficking and neurotransmission were identified and they should be incorporated into individual SVs with the correct stoichiometry to be functional for subsequent exocytosis [3, 4]. Indeed, some SV proteins including Syt1, vesicular glutamate transporter 1(vGlut1), and SV2A/B display little inter-vesicle variation, thus elaborate molecular mechanisms must exist to control the fidelity of SV protein sorting while maintaining the speed of exo-endocytosis [5]. One possibility is that exocytosed SV proteins remain clustered at the active zone and are then retrieved as a cluster during endocytosis as in the case of a specific exocytic mode called kiss-and-run [6]. Alternatively, each SV protein is precisely recruited to individual SVs by cargo-specific recognition and sorting by dedicated adaptors [7]. Indeed, recent studies have identified various cargo-specific adaptors including AP180/CALM as adaptors for vesicle associated membrane protein 2 (VAMP2; also referred to as synaptobrevin2) [8–10], and SV2A/B/stonin2 as adaptors for Syt1 [5, 11–14]. Such that each regenerated individual synaptic vesicle will obtain all essential synaptic vesicle proteins with high fidelity. In addition, recent studies indicated that the association between SV proteins is also critical for their accurate retrieval and sorting into SVs. For example, synaptophysin and SV2A/B associates with VAMP2 and Syt1, respectively, facilitating their endocytic retrieval [15, 16].

Syt1, a 65 kDa SV protein, is the major Ca^2+^ sensor for SV exocytosis. It contains two C2 domains (termed C2A and C2B) and is anchored to the membrane by a single N-terminal transmembrane domain [17, 18]. Previous studies proved that a fast synchronous component of glutamate release was mostly abolished in hippocampal neurons of Syt1 knock-out (KO) mice [17, 18]. Evidently, it is an indispensable SV cargo and previous studies showed that endocytic sorting of Syt1 is mediated by the overlapping activities of stonin2 and SV2A/B, thus suggesting that both cargo-specific, as well as the association between SV proteins, ensure proper sorting of essential Syt1 into SVs [19, 20]. Interestingly, the similar endocytic defects of Syt1 in neurons lacking either SV2A/B or stonin2 has been found, indicating that these molecules are functionally redundant for Syt1 retrieval [13, 15, 19, 20]. Considering the role of Syt1 as an exocytic regulator, such fail-safe mechanism is essential for accurate Syt1 retrieval.

The SGIP1 was first discovered by the differential display PCR analysis of hypothalamus RNA from lean and obese *P. obesus* (*Psammomys obesus*; Israeli sand rat) [21]. Targeted reduction of hypothalamic SGIP1 mRNA by infusion of antisense oligonucleotides inhibited food intake and decreased body weight in *P. obesus* [21]. Since SGIP1 interacts with endophilin, a mediator of vesicle recycling and endocytosis, it is known as an endocytic protein that has a functional role in neuronal systems in energy homeostasis [21–23]. Recent studies further identified SGIP1 as a homolog of FCHo1/2, a muniscin family member of key endocytic adaptors of CME [24–27]. The muniscin family has conserved N-terminal domain homologous to the crescent-shaped membrane-tubulating EFC/F-BAR domains and a C-terminal μHDs that interact with the endocytic adaptor/scaffold Ede1/Eps15 [28]. SGIP1 has the membrane phospholipid-binding residue in N-terminal instead of EFC-BAR domains and a C-terminal μHD [23]. SGIP1α is the brain-specific and the longest splicing variant of SGIP1 [23]. Compared to SGIP1, SGIP1α has two additional regions: an additional 28 amino acids (aa 34–61) in N-terminal, and another extra 20 amino acids (aa 550–569) in a C-terminal [23]. SGIP1α interacts with Eps15 [29], intersectin [30], and AP-2 [25] and is suggested to play a role in CME [23]. Despite its possibility, however, the functional significance of SGIP1α in the brain, especially during SV recycling, remains unknown.

In this study, we identified SGIP1α as a novel interactor of Syt1 at hippocampal neurons. We found that the C2 domains of Syt1 interact with μHD of SGIP1α and SGIP1α functions as a selective sorting adaptor for endocytic internalization and sorting of Syt1. We further found that μHD of SGIP1α is structurally similar to those of AP-2 and stonin2, which are known endocytic adaptors for Syt1. Together, we proposed the complementary fail-safe mechanism for Syt1 retrieval by SV2A/B-stonin2-SGIP1α which allows synapses to ensure the accurate sorting of Syt1 for subsequent neurotransmission.

## Materials and methods

### DNA constructs

Full-length mouse GFP- tagged SGIP1 plasmid was kindly provided by Marek Michalak (University of Alberta, Edmonton, Alberta, Canada). Recombinant human GST-Syt1-C2AB domain was kindly provided by Dr. Namgi Lee (Seoul National University, Seoul, Korea). Synaptophysin-pHluorin, VAMP2-pHluorin, and Syt1-pHluorin were provided by Dr. Leon Lagnado (Medical Research Council), Dr. J. Rothman (Sloan Kettering Cancer Center) and Dr. Volker Haucke (Leibniz Institute for Molecular Pharmacology), respectively. We generated full-length SGIP1α (NM_001285852.1) by inserting additional amino acid sequence in GFP-SGIP1 template by PCR and then ligated into the XhoI-KpnI sites in HA-C1 vector and FLAG-C1 vector. By application of PCR site-directed mutagenesis, we prepared several SGIP1 mutation constructs: HA-SGIP1α-μHD (aa 550–854), HA-SGIP1α-ΔμHD (aa 1–549) and HA-SGIP1α-mut (W771A/K781A). The fidelity of all constructs was verified by DNA sequencing. DNA constructs were purified from *E.coli* DH5α using a midi prep kit (Promega, Madison, WI) according to the instructions of the manufacturer.

### RNA-mediated interference and rescue experiments

The RNA interference (RNAi)-mediated SGIP1 KD was carried out by expressing shRNA through pU6 expression vector. Designed shRNA was cloned into the pSIREN-U6-mRFP vector (Clontech, Palo Alto, CA) for transient transfection or AAV-U6-GFP vector (Cell Biolabs, San Diego, CA) for adeno-associated virus (AAV) production. The targeted sequence of mouse SGIP1 from its cDNA sequence was 5′- GGTTCTTTACTGGCGAGATTT -3′ (nucleotides 2386–2406) common to rat SGIP1 cDNA sequence. In particular, this target sequence is also present in all five isoforms of SGIP1. The down-regulation efficiency of shRNA was examined in AAV -infected cortical neurons. To exclude the artificial effects of the expression vector, a scrambled sequence of the forward primer was designed; 5′ – GTGGCCTGTAGTTTAGTTACT – 3′ (http://www.sirnawizard.com/). The BFP-shRNA-resistant form was manipulated from GFP-tagged SGIP1α by point mutation. All constructs were verified by sequencing. After transfection, cells were cultured for more than 72 h.

### Antibodies

Mouse polyclonal anti-β-tubulin was purchased from Abcam (Cambridge, UK). Mouse monoclonal HA antibody was purchased from COVANCE (Princeton, SN). Anti-Syt1 was from Synaptic Systems (Göttingen, Germany). Anti-SGIP1 was purchased from Sigma-Aldrich (Merck, Darmstadt, Germany), HRP-conjugated anti-rabbit or anti-mouse secondary antibodies were from Jackson ImmunoResearch (West Grove, PA).

### Cell culture and transfection

Human embryonic kidney (HEK) 293 T cells (ATCC, Manassas, VA) were cultured at 37 °C, in air supplemented with 5% CO_2_ and in Dulbecco’s modified Eagle’s medium (Invitrogen, Carlsbad, CA) supplemented with 10% fetal bovine serum (GE Healthcare Life Sciences, Uppsala, Sweden). Transfection was carried out using Lipofectamine 2000 (Invitrogen), and cells were observed 36–48 h after transfection.

### Western blot

HEK293T cells transfected with plasmids encoding FLAG-SGIP1α or AAV-virus infected cortical neurons were washed three times with ice-cold PBS. Immunoblotting was performed from cell lysates generated in RIPA buffer (20 mM Tris, pH 7.5, 100 mM NaCl, 50 mM NaF, 1% SDS, 1 mM orthovanadate) supplemented with protease inhibitor cocktail (Roche, Mannheim, Germany). Protein concentrations were measured using a bicinchoninic acid (BCA) protein assay reagent kit (ThermoFisher, Waltham, MA). Protein extracts were separated by SDS-PAGE and transferred to polyvinylidene difluoride (PVDF) membranes (Pall Life Science, Ann Arbor, MI). The membranes were blocked for 1 h with 5% nonfat dry milk in TBS/T (10 mM Tris-Cl, pH 7.6, 100 mM NaCl and 0.1% Tween 20) and incubated with anti-SGIP1 (1:1000) or β-tubulin (1:1000) primary antibodies for 2 h at room temperature(RT) or overnight at 4 °C, and with corresponding HRP-conjugated secondary antibody for 1 h. Chemiluminescence reactions were performed with AbSignal Western detection kit system (AbClon, Seoul, South Korea) and detected using an ImageQuant LAS-4000 (GE Healthcare Life Sciences); images were analyzed by the ImageJ software (NIH, Bethesda, MD).

### GST pull-down assays

Recombinant human Syt1-C2AB domain with an N-terminal GST tag was overexpressed in *E.Coli* BL21. Bacterial cells were grown in 5 mL of Luria-Bertani (LB) broth at 37 °C for 2 h and subsequently inoculated to 200 mL 2xYT medium broth supplemented with 100 mg/ml Ampicillin. Cultures were grown at 37 °C for ~ 4 h with vigorous shaking and overexpression was subsequently induced by 0.25 mM isopropyl β-D-1-thiogalactopyranoside (IPTG) at 20 °C for 16 h. Bacterial cells were harvested by centrifugation at 4500 g and 4 °C for 15 min, and the pellet was resuspended with hypotonic buffer (20 mM Tris-HCl pH 8.0, 150 mM NaCl, 1 mM EGTA, 1 mM MgCl_2_) supplemented with protease inhibitor cocktail. Cells were lysed by sonication on ice and the cell extract was cleared from intact cells and debris by centrifugation at 16,000 g and 4 °C for 30 min. After centrifugation, the supernatant was incubated with GST (GE Healthcare Life Sciences) beads for the in vitro binding assay. 24–48 h post-transfection, transiently transfected HEK293T cells were lysed in ice-cold buffer (20 mM Tris-HCl pH 8.0, 137 mM NaCl, 2 mM EDTA, 1% Triton X-100, 1 mM PMSF, 0.3% inhibitor cocktail). Cleared cell extracts were incubated with GST fusion proteins on a rotator (1 mg of cell extract at 1 mg/ml) for 4 h at 4 °C. After extensive washes, bound proteins were eluted with 30 μl of SDS sample buffer. Samples were analyzed by Western blot.

### Primary neuron culture and transfection

All of the animal experiments were performed according to the Institute of animal care and use committee (IACUC) guidelines of Seoul National University. Primary rat hippocampal or cortical neurons derived from embryonic day 18 Sprague Dawley fetal rats of either sex were prepared as described previously [31]. Briefly, hippocampi or prefrontal cortexes were dissected, dissociated with papain, and triturated with a polished half-bore pasteur pipette. Approximately 2.5 × 10^5^ cells were resuspended in Hank’s Balanced Salt Solution (HBSS, GE Healthcare Life Sciences) supplemented with 0.6% glucose, 1 mM pyruvate, 2 mM L-glutamine, and 10% (v/v) FBS (GE Healthcare Life Sciences) and plated on Poly-D-lysine-coated glass coverslips in a 60-mm Petri dish. 4 h after plating, the medium was replaced with neurobasal medium (Invitrogen) supplemented with 2% (v/v) NS21, 0.5 mM L-glutamine. 4 mM 1-β-D-cytosine-arabinofuranoside (Ara-C; Sigma) was added as needed. Cortical neurons derived from prefrontal cortexes at a density of 5.0 × 10^6^ were plated on poly-D-lysine-coated 100 mm Culture dish. Neurons were transfected using a modified Ca^2+^-phosphate method. Briefly, 6 μg of DNA and 9.3 μl of 2 M CaCl_2_ were mixed in distilled water to a total volume of 75 μl and the same volume of 2xBBS (50 mM BES, 280 mM NaCl, and 1.5 mM Na_2_HPO_4_, pH 7.1) was added. The cell culture medium was completely replaced by transfection medium (MEM; 1 mM sodium pyruvate, 0.6% glucose, 10 mM HEPES, 1 mM Kynurenic acid, and 10 mM MgCl_2_, pH 7.71), and the DNA mixture was added to the cells and incubated in a 5% CO_2_ incubator for 60 min. Cells were washed twice with a washing medium (pH 7.30), and then returned to the original culture medium. Neurons were co-transfected at *days* in vitro (DIV) 8–9 and analyzed at DIV 16–21. Co-transfection was performed at a ratio of 1:2 and triple-transfection at a ratio of 1:1:1.

### Immunocytochemistry and image acquisition

Cultured neurons were fixed in 4% (V/V) paraformaldehyde, 4% (V/V) sucrose in PBS for 15 min and subsequently permeabilized with 0.25% (V/V) Triton X-100 in PBS for 3 min at RT. Neurons were then blocked with 10% bovine serum albumin (BSA) in PBS for 1 h at RT. Primary antibodies diluted 1:500 in 3% (V/V) BSA in PBS were added and incubated for 2 h at RT. Secondary antibodies were diluted 1:2000 in 3% (V/V) BSA in PBS and incubated for 1 h at RT. Immunofluorescence labeled cells were mounted and analyzed using Olympus IX-71 inverted microscope with 40X, 1.35 NA lens, an EMCCD camera (iXon897, Andor Technologies, Belfast, Northern Ireland) and MetaMorph Imaging software (Molecular Devices, San Jose, CA).

### pHluorin endocytosis assay and image analysis

Coverslips were mounted in a perfusion/stimulation chamber equipped with platinum-iridium field stimulus electrodes (Chamlide; LCI, Korea) on the stage of an Olympus IX-71 inverted microscope. The cells were continuously perfused at 34 °C with tyrode solution containing the following: 136 mM NaCl, 2.5 mM KCl, 2 mM CaCl_2_, 1.3 mM MgCl_2_, 10 mM HEPES, and 10 mM glucose, pH 7.3. The temperature of the chamber and lens was controlled by a heating controller system (LCI). 10 μM 6-cyano-7-nitroquinoxaline-2,3-dione (CNQX) and 50 μM of DL-2-amino-5-phosphonovaleric acid (AP-V) were added to imaging buffer to reduce spontaneous activity and to prevent recurrent excitation during stimulation. Time-lapse images were acquired every 5 s for 5 min using a back-illuminated Andor iXon 897 EMCCD camera driven by MetaMorph Imaging software. From the fifth frame, the cells were stimulated (1 ms, 20–50 V, bipolar) using an A310 Accupulser current stimulator (World Precision Instruments, Sarasota, FL). Quantitative measurements of the fluorescence intensity at individual boutons were obtained by averaging the pixel intensities of the selected area using ImageJ. Individual regions were selected by hand and regions of interest were drawn around the synaptic boutons, then average intensities were calculated. Large puncta which typically represent clusters of smaller synapses were rejected during the selection process. The center of each synapse was calculated to compensate for any image shift over the course of the experiment. Fluorescence was expressed in intensity units that correspond to fluorescence values averaged over all pixels within the region of interest. Net fluorescence changes, for individual boutons, were obtained by subtracting the average intensity of the first four frames (F_0_) from the intensity of each frame (F_t_). They were then normalized to the maximum fluorescence intensity (F_max_ − F_0_) and averaged. All fitting was done using individual error bars to weight the fit using Origin Pro 9.0 (Origin Lab Corporation, Northampton, MA). To obtain the endocytic time constant after stimulation, the decay of pH-probe after stimulation was fitted with a single exponential function. Data were collected from ~ 140 boutons of 12–24 neurons in each coverslip and “n” stands for the number of coverslips. Statistical analysis was performed with GraphPad Prism 7.0 (GraphPad Software, San Diego, CA). Once the normality assumption was satisfied, two independent groups were compared by Student’s two sample *t*-test.

### Surface fraction measurement

For a surface fraction of Syt1-pHluorin measurement cultured hippocampal neurons were transfected with mRFP-tagged shRNA targeting SGIP1α or scrambled RNA and Syt1-pHluroin. Transfected neurons were mounted in closed perfusion/stimulation chamber (LCI) and perfused continuously with standard pH 7.4 tyrode solution or non-permeable pH 5.5 acid solution at RT by using MPS-8-multi-valve perfusion system (LCI) at 0.5 mL/min. 300 action potentials (APs) at 10 Hz were applied using platinum-iridium electrodes and A310 Accupulser current stimulator (World Precision Instruments). Fluorescent images of Syt1-pHluorin were acquired at every 5 s and analyzed using ImageJ through marking individual rectangular regions of interest (ROIs) by hand.

### Modeling of crystal structures and multiple sequence alignments

Ribbon models of the crystal structures were obtained from SWISS-MODEL https://swissmodel.expasy.org/ Accessed 23 July 2018 [32]. Multiple protein sequence alignments were performed using the Constraint-based Multiple Alignment Tool https://www.ncbi.nlm.nih.gov/tools/cobalt/ Accessed 3 March 2019.

## Results

**Fig. 1:**
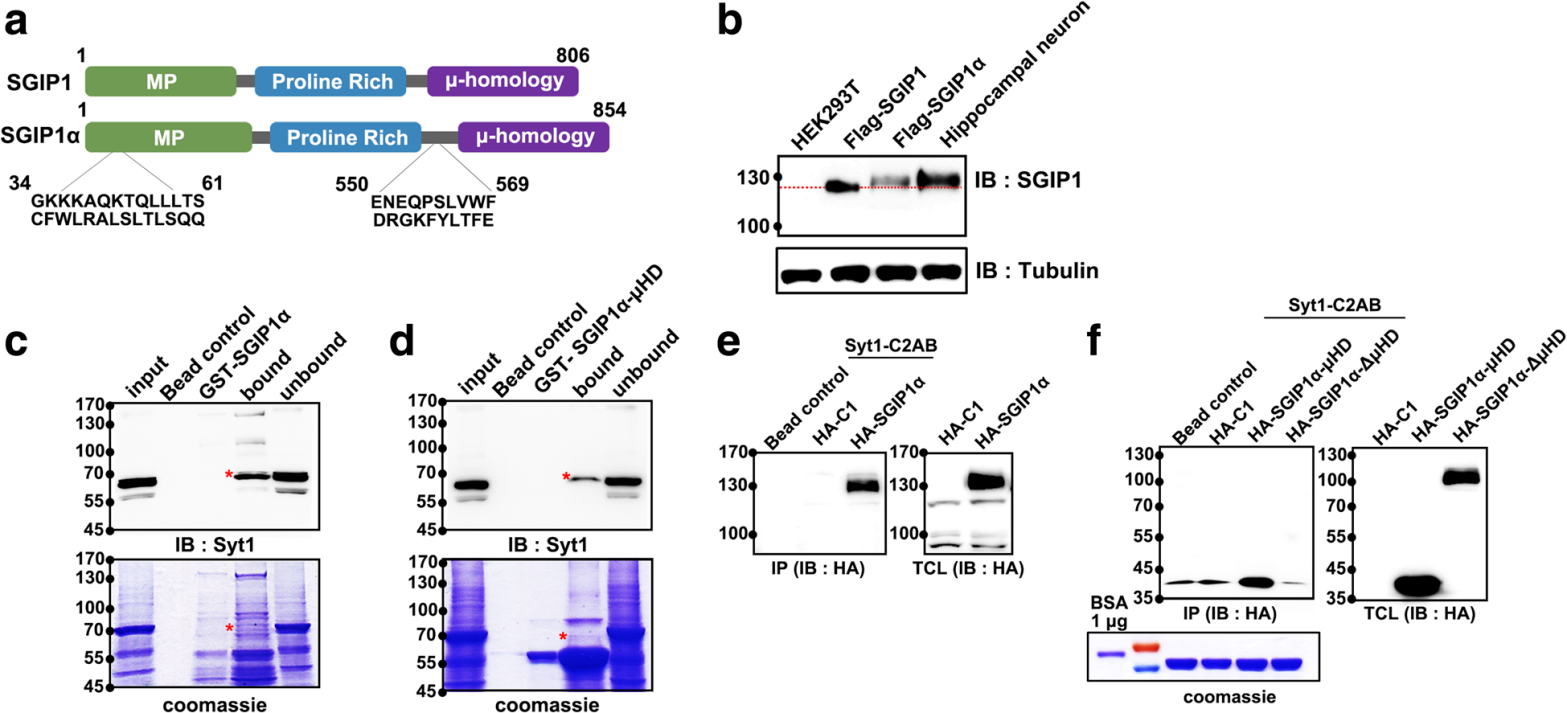
SGIP1α is expressed in cultured primary neurons and binds to Syt1. **a** Domain structures of SGIP1 and SGIP1α. SGIP1α contains additional 28 amino acids in the MP domain and 20 amino acids between the PRD and μHD. **b** Whole cell lysates from HEK293T, HEK293T with FLAG-SGIP1 and FLAG-SGIP1α and cultured hippocampal neurons were resolved by SDS-PAGE and transferred to nitrocellulose and immunoblotted with SGIP1 antibody. The red dotted line indicates the size of FLAG-SGIP1. **c, d** Hippocampal neuron lysate was pull-downed with purified GST-SGIP1α (**c**) or purified GST- SGIP1α-μHD (**d**) immobilized on glutathione-sepharose. Bound proteins were eluted from the beads by boiling and resolved by SDS-PAGE and either stained with Coomassie blue or transferred to nitrocellulose. Replicate blots were probed with Syt1 antibody as indicated. The asterisk indicates the band of Syt1. **e** Purified GST-Syt1-C2AB fusion protein immobilized on beads were incubated with cell extracts derived from HEK293T cells transfected with HA-C1 or HA-SGIP1α. Bound proteins were detected by immunoblotting for HA. **f** GST-Syt1-C2AB fusion protein immobilized on beads were incubated with cell extracts derived from HEK293T cells transfected with HA-C1 or HA-SGIP1α-μHD or HA- SGIP1α ΔμHD. Bound proteins were detected by immunoblotting for antibody against HA. IB, Immunoblot; IP, Immunoprecipitation; TCL, Total cell lysates

The previous study has identified SGIP1α as a brain-specific longest isoform of SGIP1 [23], and we first analyzed the expression of SGIP1α in primary cultured rat hippocampal neurons. Since there is no specific antibody that distinguishes SGIP1α from SGIP1, we compared the size of endogenous SGIP1α in hippocampal neurons with that of FLAG-tagged SGIP1α or SGIP1 transfected in the HEK293T cells with a commercial antibody against SGIP1. We found that hippocampal neurons endogenously expressed a protein with a molecular mass of about 130 kDa, which was comparable to the size of FLAG-tagged SGIP1α but higher than that of FLAG-tagged SGIP1 in HEK293T (Fig. 1b). These results suggest that SGIP1α, not SGIP1 is endogenously expressed in hippocampal neurons.

### SGIP1α physically interacts with Syt1

To identify the binding partners of SGIP1α in neurons, we performed GST-pull down assay. The purified GST-tagged SGIP1α were incubated with rat hippocampal neuron lysates. Fraction bound or unbound with SGIP1α were resolved by SDS-PAGE and stained with Coomassie blue. Multiple bands with varying molecular weights were detected although some of them appeared to be non-specific when we compared them to bands in GST-SGIP1α lane (the lane without neuron lysate; Fig. 1c). Four bands were identified as specific, which include one at 130 kDa (which seems to be EPS15 [29]), two around 65 kDa and one below 55 kDa (Fig. 1c). Given the proposed role in endocytosis and brain-specific expression pattern, we reasoned that SGIP1α could interact with a presynaptic protein expressed in SVs. We noticed a particular band around 65 kDa since we are aware that the prominent SV resident protein, Syt1 has a molecular weight of 60~70 kDa. Indeed, the band was confirmed as Syt1 by western blot with a specific antibody against Syt1 (Fig. 1c).

To find out which domain of SGIP1α interacts with Syt1, we looked the published literatures up and found that Syt1 interacts with the μHD of AP-2 via its C2 domains [33, 34]. Since SGIP1α also contains μHD, it would be the domain most likely to interact with Syt1. Indeed, when the same experiment was performed with μHD of SGIP1α, the band with identical size was detected thus indicating that SGIP1α binds with endogenous Syt1 via its μHD.

To corroborate the association of SGIP1α with Syt1, we conducted GST-pull down assay experiments. The GST-fused cytoplasmic (C2AB) domains of Syt1 were purified and incubated with the transfected HEK293T lysates. As shown in Fig. 1e, HA-tagged SGIP1α bound to the C2AB fusion protein while it did not bind to GST control beads. When the same experiment was carried out with μHD (aa 550–854) and ΔμHD domain (aa 1–549) of SGIP1α, the only μHD bound to C2AB, suggesting the interaction between C-terminal μHD domain of SGIP1α and C2 domains of Syt1.

**Fig. 2:**
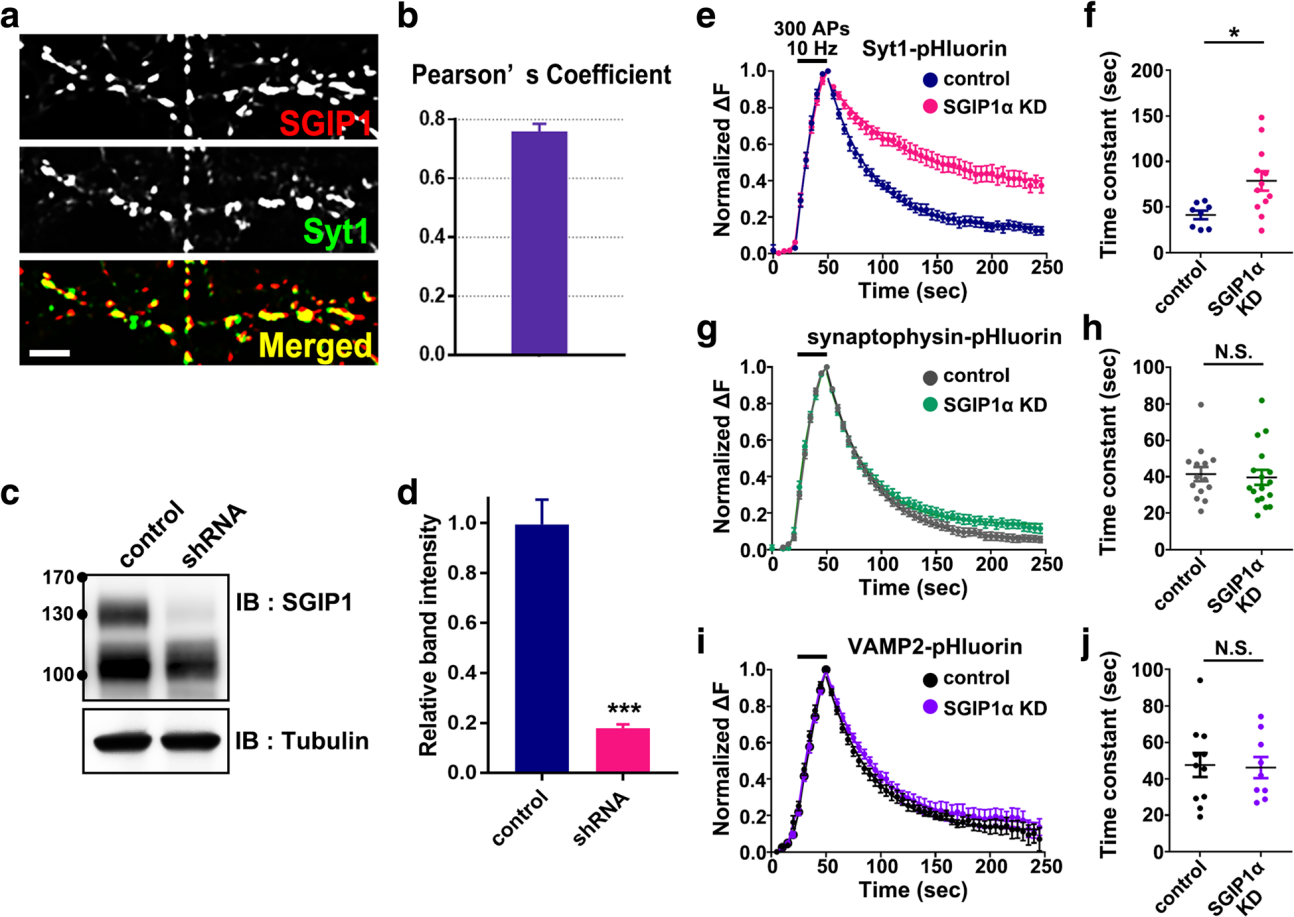
SGIP1α is expressed at the presynaptic terminals of cultured hippocampal neurons and SGIP1α KD causes the selective defects in the internalization of Syt1. **a** Rat brain hippocampal neurons were fixed at DIV 21 and immunostained with specific antibodies against SGIP1α (red) and Syt1 (green). Scale bar, 5 μm. **b** Pearson’s coefficient of colocalization between SGIP1α and Syt1 (*n* = 10). (**c** and **d**) Primary cultured neurons at DIV 6 were infected with AAV-shRNA-SGIP1α, and the KD efficiency was confirmed by western blotting with anti-SGIP1 antibody at DIV 21 (*n* = 3 blots). **e-j** Average Syt1-pHluorin (**e**, control: *n* = 8, KD: *n* = 12), synaptophysin-pHluorin (**g**, control: *n* = 14, KD: *n* = 17), or VAMP2-pHluorin (I, control: *n* = 11, KD: *n* = 19) fluorescence intensity profiles in neurons expressing empty vector or shRNA-SGIP1α, plotted as ΔF/F_0_ against time, after stimulation with 300 APs at 10 Hz (dark bar). Fluorescence values were normalized to the maximal fluorescence signal in each experimental condition. **f, h, j** τ values for the decay of Syt1-pHluorin (**f**), synaptophysin-pHluorin (**h**), or VAMP2-pHluorin (**j**) after stimulation fitted by a single exponential. Data are means ± SEM; *p < 0.05, ***p < 0.001 by Student’s *t*-test, N.S., not significant

To gain insight into the possible function of SGIP1α in hippocampal neurons, we immunostained primary cultured hippocampal neurons at DIV 21 with specific antibodies against endogenous SGIP1α and Syt1. Consistent with their physical association, approximately 76% of SGIP1α colocalized with Syt1 at synapses in cultured neurons (Fig. 2 a and b). The experiment revealed that neuronal-specific isoform SGIP1α is expressed in the presynaptic terminals of primary cultured neurons where it colocalizes with Syt1.

### SGIP1α KD selectively impairs the internalization of Syt1 but not synaptophysin or VAMP 2 from the neuronal surface

To assess the physiological effects of SGIP1α, we generated an AAV-shRNA construct targeting SGIP1α. Suppression of SGIP1α expression level by shRNA was confirmed by western blot analysis of AAV-shRNA infected cultured cortical neurons (Fig. 2 c and d).

In primary cultured hippocampal neurons, SV proteins including Syt1 are constitutively recycled between SVs and the presynaptic plasma membrane. To quantitatively estimate the trafficking of Syt1 between vesicular and plasma pools, we used Syt1-pHluorin, which is a fusion construct between the luminal domain of Syt1 and pHluorin, a modified GFP with high pH sensitivity [35–37] such that its fluorescence is quenched in acidic conditions and increased in basic conditions upon exocytosis to the extracellular space. The exocytosis was evoked by applying 10 Hz field stimulus for 30 s. The Syt1-pHluorin fluorescence intensity of the individual boutons increased rapidly and decayed after reaching a peak with an exponential time course, revealing the kinetics of endocytosis.

We found that in SGIP1α KD boutons, Syt1 endocytosis was significantly slowed with a longer time constant than in control boutons (τ = 41.25 ± 4.67 s for the control; τ = 78.63 ± 10.76 s for SGIP1α KD; Fig. 2 c e and f). When we repeated endocytosis assays using other SV proteins, synaptophysin (Fig. 2 g and h) or VAMP2 (Fig. 2) i and j, however, no endocytic defects were observed (synaptophysin-pHluorin: τ = 41.44 ± 3.91 s for the control; τ = 39.63 ± 4.12 s for SGIP1α KD; VAMP2-pHluorin: τ = 47.57 ± 6.58 s for the control; τ = 46.17 ± 5.85 s for SGIP1α KD). These results suggest that SGIP1α KD hinders the endocytosis of Syt1, but not synaptophysin or VAMP2.

**Fig. 3:**
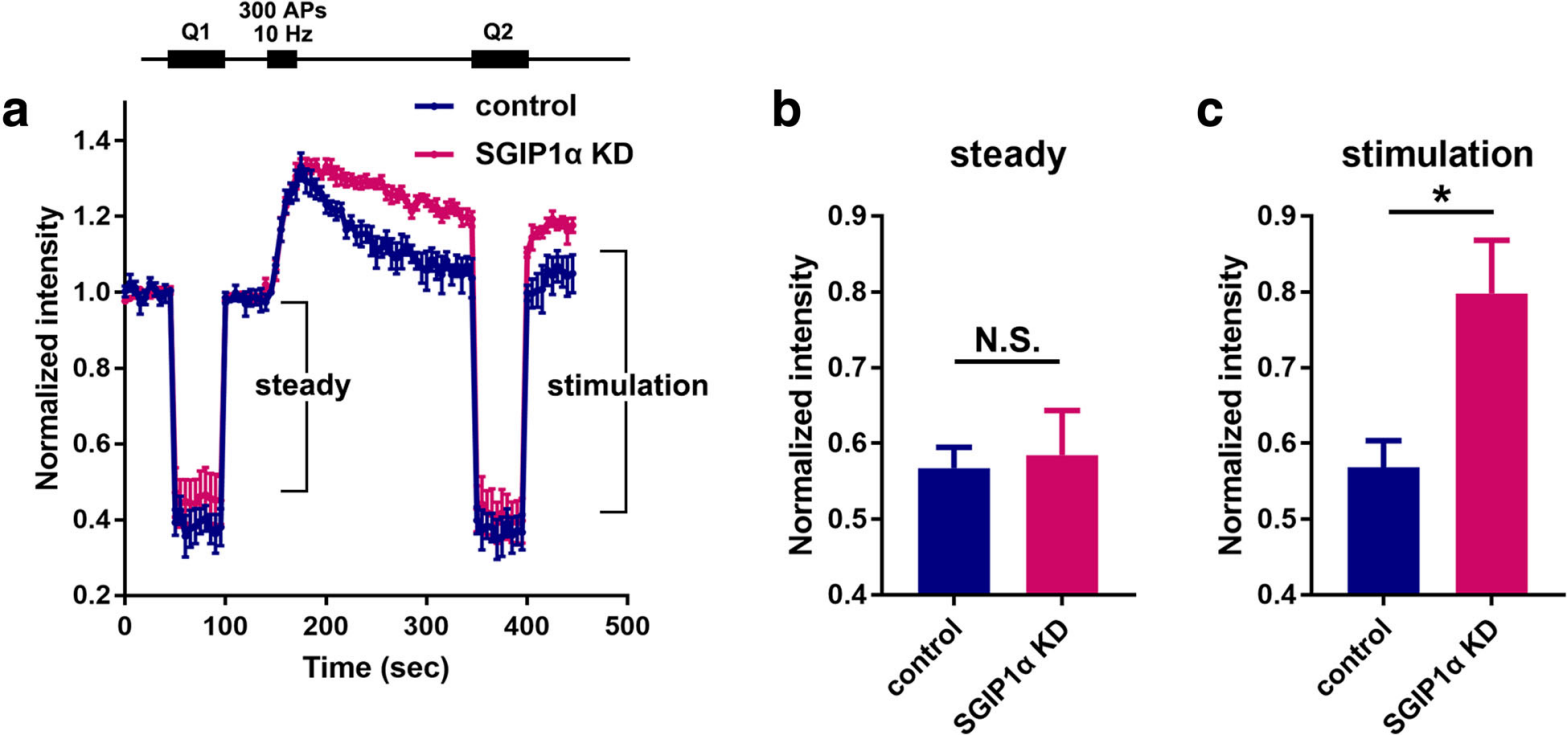
**a** Average traces of Syt1-pHluorin signal in control and SGIP1α KD during pH exchange experiments. Non-permeable pH 5.5 acid solution (Q1 and Q2 period) rapidly quenches all surface Syt1-pHluorin. After 50 s, the solution was changed back to pH 7.4. After 300 APs at 10 Hz, the extracellular solution was changed again to pH 5.5 for 50 s then back to pH 7.4. Net Syt-pH fluorescence changes were normalized to the initial values for individual experiments and averaged. Steady and stimulation represents the surface fraction of Syt1-pHluorin probe before and after stimulation, respectively. **b**, **c** Bar graphs indicate the surface fraction of Syt1-pHluorin probe before stimulation (**b**, control, 0.58 ± 0.03, *n* = 4; KD, 0.58 ± 0.06, *n* = 4) and after stimulation (**c**, control, 0.57 ± 0.03, *n* = 4; KD, 0.80 ± 0.07, *n* = 4). Data are presented as mean ± SEM. *p < 0.05

We then quantitatively analyzed the effect of SGIP1α KD on the partitioning of Syt1 between the presynaptic plasma membrane and internal SV pool in primary hippocampal neurons (Fig. 3). To assess the surface and vesicular pools of Syt1-pHluorin under steady state and stimulating conditions, the relative fluorescence values were quantitatively measured using the acid quenching-dequenching protocol [11] before and after stimulation with 300 APs (Fig. 3 a). We found that under resting conditions, SGIP1α KD did not induce a significant change in the relative plasma membrane to the vesicle fraction of Syt1 (Fig. 3 a and b). However, the relative plasma membrane fraction of Syt1-pHluorin at presynaptic boutons increased significantly after stimulation with 300 APs (Fig. 3 a and c). These results indicated that the surface stranded portion of Syt1 is increased only after stimulation but not under resting conditions in KD neurons.

### Structural similarity of SGIP1α μHD with those of AP-2 μ and stonin2

The μHD is an ~ 280 amino acid residue protein-protein interaction module found in endocytic proteins involved in CME such as AP complexes, stonins, and proteins of the muniscin family including Syp1, FCHO1/2, and SGIP1 [26, 27, 38]. The μHD of the muniscin proteins is involved in the interactions with the endocytic adaptor-scaffold proteins Ede1-Eps15 [38]. We observed that SGIP1α associates with the Syt1 C2 domains via its μHD (Fig. 1f). To further delineate the interaction between μHD of SGIP1 and Syt1, we generated a molecular homology model of SGIP1α μHD using AP-2 μ and stonin2- μHD as templates.

**Fig. 4:**
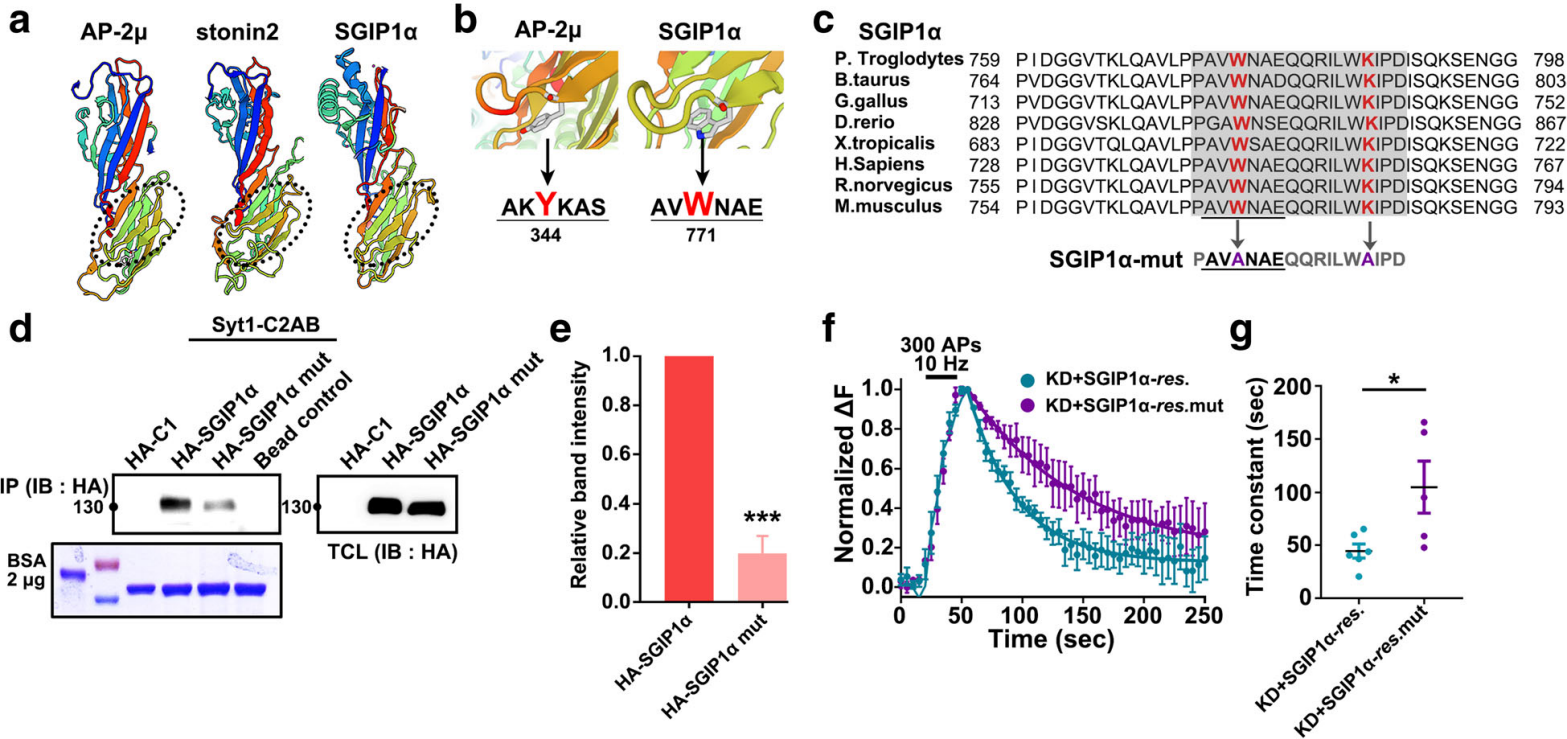
SGIP1α mutant W771A/K781A binds less efficiently to Syt1 and fails to rescue the endocytic defect of Syt1 caused by SGIP1α KD. **a** Ribbon models of the crystal structures of AP-2 μ subunit, μHD of stonin2 and SGIP1α. The β-sheets of the μHDs are colored lime green and circled in dotted line. **b** An enlarged representation of the μHDs of AP-2 and SGIP1α. The μHDs and peptides are shown as ribbon models and sticks, respectively. Comparing overall structural similarity, W771 of SGIP1α was identified as the residue corresponding to Y344 of AP-2. **c** Multiple protein sequence alignment of SGIP1α and orthologues from chimpanzee, cattle, chicken, zebrafish, frog, human, rat, mouse. Numbers refer to the amino acid residue within the predicted β strand. Conserved residues are colored in red. (below) Corresponding sequences of WT SGIP1α and a double mutant of SGIP1α W771A/K781A (SGIP1α-mut). **d** Purified GST-Syt1-C2AB fusion protein immobilized on beads were incubated with cell extracts derived from HEK293T cells transfected with HA-C1 or HA-SGIP1α or HA-SGIP1α mutant W771A/K781A. Bound proteins were detected by immunoblotting for antibody against HA. Coomassie blue staining of the gels revealed that similar amounts of GST fusion proteins were used. **e** Relative band intensity of bound Syt1 (1 ± 0 for SGIP1α, 0.20 ± 0.07 for SGIP1α-mut, *n* = 3 independent blots). **f** Average Syt1-pHluorin fluorescence intensity profiles in neurons expressing shRNA-resistant SGIP1α (KD + SGIP1α, *n* = 6) or shRNA-resistant SGIP1α-W771A/K781A (KD + SGIP1α mut, *n* = 5) with shRNA-SGIP1 and Syt1-pHluorin after stimulation with 300 APs at 10 Hz (dark bar). Fluorescence values were normalized to the maximal fluorescence signal in each experimental condition. **g** τ values for the decay of fluorescence fitted by a single exponential (KD + SGIP1α-*res*.: *n* = 6, KD + SGIP1α- *res.*mut: *n* = 5). Data are presented as means ± SEM; *p < 0.05, ***p < 0.001 by Student’s *t*-test

SGIP1α μHD displays an overall β-fold structure very similar to that of AP-2 μ and stonin2-μHD (Fig. 4 a). The β strand is suggested to harbor the Syt1 binding site with AP-2 μ containing bulky aromatic residue. SGIP1α also has a bulky aromatic tryptophan residue, Trp771, which corresponds to Tyr344 in AP-2 μ, and Tyr784 in human stonin2 (Fig. 4 b). Indeed, Trp771, as well as several flanking residues, is also evolutionarily conserved within SGIP1 family members of vertebrates, suggesting its functional importance (Fig. 4 c). Thus, it could be the residue that has Syt1 binding affinity.

### SGIP1α mutant W771A/K781A binds less efficiently to Syt1 and fails to rescue the endocytic defect of Syt1 by SGIP1α KD

The previous study found that Tyr344A/Lys354A mutant of AP-2 μ bound less efficiently to Syt1 than wild-type (WT) (~ 20% of WT) [33]. Given the homology between AP-2 μ and SGIP1α μHD, we hypothesized that a double mutant of SGIP1α, in which Trp771 and Lys781 that correspond to Tyr344 and Lys354 of AP-2 μ mutated to alanine (Fig. 4 c), would have a lower affinity for Syt1. We transfected HEK293T with SGIP1α or a W771A/K781A mutant and performed GST-pull down experiments using GST-fused Syt1. As expected, the affinity of W771A/K781A mutant for Syt1-C2AB was substantially reduced compared to that of the WT SGIP1α (Fig. 4 d and e).

We then expressed the shRNA-resistant form of SGIP1α or its W771A/K781A mutant under the conditions of SGIP1α KD to test whether it could rescue KD-mediated defects in Syt1 internalization. We found that SGIP1α WT completely rescued the defects in Syt1 internalization, but the W771A/K781A mutant did not (τ = 44.59 ± 6.60 s for the WT over SGIP1 KD; τ = 104.8 ± 24.32 s for SGIP1α mutant over KD; Fig. 4 e and f). These results ultimately suggest that the Trp771/Lys781 residues in the μHD of SGIP1α are critical for the binding and internalization of Syt1.

## Discussion

In this study, we provide multiple experimental evidence for a cargo-selective role of SGIP1α in the retrieval of Syt1 on the neuronal surface. Besides its originally identified function as a candidate gene for regulating energy balance and potentially important in determining obesity risk in humans [21, 22], several studies have also found that SGIP1 is an endocytic protein that functions as the molecular interface between the basic machinery of endocytosis and components of actin cytoskeletal rearrangements [23, 24]. More recently, a brain-specific and the longest splicing variant SGIP1α has been identified and it is known to play an essential role in CME by interacting with the Eps15 and AP-2 complexes as well as by deforming the plasma membrane [23]. Its functional significance especially during SV recycling, however, has not been studied.

Regardless of the endocytosis mode, the newly retrieved SVs should contain essential SV proteins with the correct stoichiometry that can be functional for the next round of exocytosis. Various adapter proteins that selectively recognize individual cargoes recruit SV proteins to the newly created SVs [6]. These adapters can bind directly to cargo proteins and recruit clathrin coat proteins at the same time. Some SV cargoes, such as vGlut1, VAMP2, and Syt1, contain several retrieval motifs that are recognized by multiple different adapter proteins [39]. As the SV membrane is reformed at various rates through different modes depending on neural activity, a “safeguard” system with multiple motifs in individual cargoes definitely help to maintain the exact stoichiometry of the essential SV cargoes required per SV [39]. On this account, a distinct pathway for SV recycling called kiss-and-run has been proposed as an alternative endocytic mode in neurons to satisfy the sorting accuracy at the appropriate rate of exo-endocytosis [40].

As a major Ca^2+^ sensor for SV exocytosis, Syt1 should be incorporated into SVs during endocytosis. Previous studies have shown that stonin2 recognizes the basic patch within the Syt1 C2 domains and interacts with Syt1 to facilitate sorting of Syt1 into retrieved SVs [11, 37]. Nevertheless, another study suggested that Syt1 mutants with reduced affinity for stonin2 are still internalized, suggesting that additional determinants and/or cooperative effects may be involved [37]. Indeed, the loss of other SV protein, SV2A/B, in mice is known to cause the defects in sorting Syt1 [20, 41]. SV2A/B deficiency impairs the efficacy of stimulation-evoked SV exocytosis, a phenotype associated with the loss of Syt1 in SVs [11]. Interestingly, the combined deficiency of SV2A/B and stonin2 causes additive defects in Syt1 sorting at the synapses [19], thus despite their distinct molecular features, they appear to perform a shared overlapping function on sorting of Syt1 in synapses. In this study, we found that SGIP1α is another selective sorting adaptor for endocytic internalization and recycling of Syt1. Our result showed that SGIP1α is not required for the retrieval of other SV proteins, synaptophysin or VAMP2 (Fig. 2), which is consistent with the previous study in which SV2A/B deficiency selectively impairs endocytic retrieval of Syt1 rather than synaptophysin [19].

It is noteworthy that the endocytosis of Syt1 is partially but not completely impaired in SGIP1α depleted neurons (Fig. 2 e and f). Indeed, the previous study also showed that deletion or KD of either SV2A/B or stonin2 results in partial missorting of Syt1 to the neuronal surface, implying the existence of functional redundancy among related proteins. Functional redundancy in a set of proteins is not rare in nervous system, especially in the SV recycling process. It could provide a protective mechanism to prevent a life-threatening disaster from happening even if only one protein is depleted. In addition, considering the fact that a single SV contains multiple copies of Syt1 (15.2 copies/vesicle) [4], we believe that SGIP1α together with SV2A/B and stonin2 may play compensatory function, rather than compete for the trafficking of Syt. We, however, cannot fully rule out the possibility in which these three proteins may function in Syt1 retrieval process at distinct steps or different activity states. In fact, the direct binding of Syt1 to AP-2 is Ca^2+^-dependent while stonin2 bind to Syt1 in a Ca^2+^-independent manner. Furthermore, the N-terminus of SV2A is required for binding to the C2B domain of Syt1 but whether this binding is Ca^2+^-dependent or not is currently unclear [42]. Therefore, although AP-2, stonin2 and SV2A/B all bind to the C2 domains of Syt1, their Ca^2+^-dependency for binding seem different. Whether SGIP1α bind to Syt1 in a Ca^2+^-dependent manner or not would certainly be of interest.

We further found the structural similarity between μHD of SGIP1α with those of stonin2 and AP-2 and showed their functional similarity within the corresponding residues (Fig. 4 a and b). We demonstrated that Trp771 residue within the β strand in the μHD of SGIP1α corresponds to the Syt1-binding Tyr344 of AP-2 μ or Tyr784 of stonin2. Evidently, a double mutant of Trp771A/Lys781A of SGIP1α not only showed reduced ability to bind to Syt1 but also could not restore the defects of Syt1 retrieval by SGIP1 KD (Fig. 4 d to g).

SV release occurs at several modes that are dependent (synchronous, asynchronous) or independent (spontaneous) on APs. Syt1 functions as a Ca^2+^ sensor at the synchronous mode, but the Ca^2+^ sensor has not been identified at the other two modes. A recent study found that the cytoplasmic protein known as Doc2 (double C2 domain) is required for spontaneous release and promotes membrane fusion in response to an exceptionally low increase in Ca^2+^ [43]. Doc2 is more sensitive to Ca^2+^ and competes with Syt1 for SNARE-complex binding during membrane fusion, thus acting as a high-affinity Ca^2+^ sensor for spontaneous release [43]. Therefore, in the presence of Doc2, normal spontaneous release and unaltered steady state SV recycling could be maintained even under SGIP1α depleted condition. In addition, Syts constitute a family of proteins and in vertebrates, 16 isoforms of Syt have been identified [44]. Besides the most abundant isoform Syt1, Syt2 is also localized to SVs and function as a Ca^2+^-sensor although they are differentially expressed in a largely non-overlapping pattern [34, 45, 46]. Other Syts including Syt3, 6 and 7 are also known to perform complementary functions in exocytosis. Whether SGIP1α interacts with Doc2 or other Syt isoforms in a manner different from Syt1 is certainly a matter of interest and further study is needed.

Together, our data clearly demonstrate that SGIP1α functions as a selective adaptor for Syt1 for its endocytic retrieval and sorting into SVs. Thus we propose the compensatory fail-safe mechanism in which SGIP1α joins a group of proteins with stonin2 and SV2A/B, allowing synapses to ensure the accurate sorting of Syt1 for subsequent neurotransmission.

## Abbreviations

APs: Action potentials
AP-V: DL-2-amino-5-phosphonovaleric acid
AZ: Active zone
BCA: Bicinchoninic acid
BSA: Bovine serum albumin
Ca^2+^: Calcium
CME: Clathrin-mediated endocytosis
CNQX: 6-cyano-7-nitroquinoxaline-2,3-dione
DIV: Days in vitro
F: Fluorescence
FCHo: Fer/Cip4 homology domain-only
HBSS: Hank’s Balanced Salt Solution
HEK: Human embryonic kidney
IACUC: Institute of animal care and use committee
IB: Immunoblot
IP: Immunoprecipitation
KD: Knock-down
KO: Knock-out
LB: Luria-Bertani
PVDF: Polyvinylidene difluoride
RNAi: RNA interference
ROIs: Regions of interest
RT: Room temperature
SGIP1: SH3-containing GRB2-like protein 3-interacting protein 1
shRNA: small hairpin RNA
SV: Synaptic vesicle
SV2A/B: Synaptic Vesicle Glycoprotein 2A/B
Syt1: Synaptotagmin 1
TCL: Total cell lysates
VAMP2: Vesicle associated membrane protein 2
vGlut1: Vesicular glutamate transporter 1
μHD: μ-homology domain
